# Political and Governance Challenges to Achieving Global HIV Goals with Injecting Drug Users: The Case of Pakistan

**DOI:** 10.15171/ijhpm.2018.131

**Published:** 2019-01-22

**Authors:** Hina Khalid, Ashley M. Fox

**Affiliations:** ^1^Department of Economics, School of Humanities and Social Sciences, Information Technology University, Lahore, Pakistan.; ^2^Rockefeller College of Public Affairs and Policy, University at Albany, Albany, NY, USA.

**Keywords:** Injecting Drug Users, HIV/AIDS, Governance, Political Commitment, South Asia

## Abstract

**Background:** The Joint United Nations Programme on HIV/AIDS (UNAIDS) has recently set the ambitious "90-90-90 target" of having 90% of people living with HIV (PLHIV) know their status, receive antiretroviral therapy (ART), and achieve viral suppression by 2020. This ambitious new goal is occurring in a context of global "scale-down" following nearly a decade of heightened investment in HIV prevention and treatment efforts. Arguably international goals spur action, however, setting unrealistic goals that do not take weak health systems and variations in the nature of the epidemic across countries into consideration may set them up for failure in unproductive ways that lead to a decline in confidence in global governance institutions. This study explores how policy actors tasked with implementing HIV programs navigate the competing demands placed upon them by development targets and national politics, particularly in the current context of waning international investments towards HIV.

**Methods:** To examine these questions, we interviewed 29 key informants comprising health experts in donor organizations and government employees in HIV programs in Pakistan, a country where HIV programs must compete with other issues for attention. Themes were identified inductively through an iterative process and findings were triangulated with various data sources and existing literature.

**Results:** We found both political and governance challenges to achieving the target, particularly in the context of the global HIV scale-down. Political challenges included, low and heterogeneous political commitment for HIV and a conservative legal environment that contributed towards a ban on opiate substitution therapy, creating low treatment coverage. Governance challenges includedstrained state and non-governmental organization (NGO) relations creating a hostile service delivery environment, weak bureaucratic and civil society capacity contributing to poor regulation of the health infrastructure, and resource mismanagement on both the part of the government and NGOs.

**Conclusion:** Our findings suggest that in a context of waning international attention to HIV, policy actors on the ground face a number of practical hurdles to achieving the ambitious targets set out by international agencies. Greater attention to the political and governance challenges of implementing HIV programs in low- and middle-income countries (LMICs) could help technical assistance agencies to develop more realistic implementation plans.

## Background

The Joint United Nations Programme on HIV/AIDS (UNAIDS) has set a number of goals since the early years of the HIV epidemic including the 3 by 5 initiative (get 3 million people on antiretrovirals [ARVs] by 2005),^1^ the 15 by 15 target (getting 15 million people on treatment by 2015)^2^ and the Getting to Zero strategy (zero new infections by 2015).^3^ More recently, UNAIDS has set an ambitious goal of having 90% of all people living with HIV (PLHIV) to know their status, receive antiretroviral therapy (ART), and achieve viral suppression by 2020, otherwise known as the 90-90-90 target.^4^ This ‘treatment as prevention’ approach is being emphasized because of recent evidence of reduced risk of transmission from individuals who are virally suppressed.^5^

To meet this new target many countries will have to rapidly scale-up HIV service delivery in an environment where there is a decline in donor funding for HIV.^6^ Following an era of high HIV funding, with the largest annualized percentage change recorded between 2000-2010, development assistance for HIV is being scaled down. Between 2014 to 2015, donor funding for HIV decreased by 13% ($8.62 to $7.53 billion).^6^ The decline in donor funding is the outcome of several interrelated factors: an appreciation of the dollar, accompanied by a depreciation of donor currencies; a delay in contributions by the United States, the largest contributor towards development assistance for HIV; and front-loaded contributions to the Global Fund by donors.^6^ In spite of scale-down, in low- and middle-income countries (LMICs), HIV continues to remain one of the most heavily funded sectors. The ratio of government spending to development assistance for HIV in these countries is twenty times higher as compared to the ratio of development assistance for health to government spending.^7^ Therefore, PLHIV in LMICs, will be among those who are the hardest hit by these funding cuts as there is heavy reliance on development assistance for funding HIV programs, making these countries susceptible to fluctuations in development assistance.^7^

While HIV funding remains an important source of revenue for health systems in LMICs, previous research has revealed the perverse effects stemming from the decade long donor infusions of HIV specific funding. These include the vertical nature of HIV funding, which focused narrowly on getting “drugs into bodies”^8^ without attention to the broader need to strengthen health systems to deliver care and primary healthcare.^9-12^ Likewise, the exceptional nature of the HIV response led to a misalignment between the actual health needs of countries and the AIDS-specific funding provided to LMICs, often leading countries to position themselves as suffering from HIV at the expense of other, more pressing health concerns.^9^ Although prominent studies have found that HIV funding has not necessarily “crowded out” donors’ support for other health programs^13^ due to overall increases in global aid,^14^ there is increasing evidence that the narrow, vertical basis of HIV funding has created unintended consequences that have weakened already fragmented health systems.^15^ For instance, HIV-specific funding has been argued to have affected the health work force, pulling the limited number of well-trained health workers into better paid positions in AIDS non-governmental organizations (NGOs) and out of the public sector, compounding health worker shortages in LMICs.^16,17^ In addition to the misalignment of priorities precipitated by the rapid increase in dedicated HIV spending, research has identified the principal-agent problems created by the misaligned incentives between donors and local communities. Local officials, the agents, face competing pressures from donors (external principals) who provide funding for HIV interventions and the people whom they represent (local principals). While external principals prioritize HIV interventions, local principals are often more interested in addressing other pressing health and development concerns. Misalignment of priorities creates pressures on agents for implementing the global goals.^11^

While some credit global targets such as the 90-90-90 with developing common goals that spur action and channel effort among governments,^18-20^ others are more skeptical of global consensus documents that lack enforceability mechanisms. For instance, Easterly argues that the Sustainable Development Goals (SDGs) are unactionable, unquantifiable, with almost all aspects of development occupying a top priority, and no concrete commitment of funding to meet the goals.^21^ Others echo his concerns in arguing that the goals are not well developed, have contradictory trade-offs, and countries with low sustainable development indicators face a bigger challenge in achieving these goals.^22,23^ Moreover, while treatment as prevention is a promising approach to reducing HIV transmission that does not require behavior change interventions, which have been shown to be relatively ineffective, others have criticized this approach, noting that this bio-medicalization of prevention efforts has a perverse effect on HIV activism, rights-based prevention and structural interventions.^24,25^ It is also argued that people in poorer countries only have access to the cheapest cocktail drugs that can have harmful consequences. Finally, the combination of funding cuts and propagation of biomedical methods has led to a health workforce which is overworked and disillusioned.^15^

As countries embark on achieving the new 90-90-90 HIV targets, research does not fully enumerate the challenges developing countries might face in achieving these targets, and the new target’s potential impact on countries’ overall HIV response. This study aimed to explore how different policy actors were navigating the challenges posed by the evolving global HIV response in a country with a conservative climate towards HIV, a large injecting drug user (IDU) population, and competing health sector priorities.

### Setting

Pakistan is an important case to study in the context of HIV scale-down in that, like many LMICs, it represents a country with a weak domestic political commitment to HIV and a tepid policy response. While heightened global attention to HIV may have temporarily bolstered the response in an otherwise weakly committed country, the scale-down of resources is likely to affect the response even more profoundly as there is little political support for the issue.^26,27^ In contrast with countries with generalized epidemics, in Pakistan HIV prevalence remains low (less than 0.1% of the adult population), and is concentrated among IDUs.^28^ In 2011, the estimated number of IDUs in Pakistan was 46 351 and the HIV sero-prevalence among them was 37.8%.^29^ These rates are much higher than the overall global prevalence of HIV among IDUs (13.1%),^30^ and consequently, most funding and attention has gone towards addressing HIV in this highly marginalized and unorganized population. However, in the absence of global attention, this group remains highly vulnerable.

Pakistan’s HIV response is best described as tepid. There is high disease stigma,^31^ no stated commitment by heads of state regarding HIV^32^ and budgetary commitment to address the disease remains very low. In 2015, the contribution of international and domestic sources for HIV program activities was $6.36 million and $3.63 million respectively.^33^ On the policy side, needle exchange programs are prohibited by law, but are still allowed to operate in the country.^34^ However, opioid substitution therapy is not allowed in the country.^35^ In terms of offering protection to PLHIV in terms of employment and discrimination, Sindh, is the only province that has currently passed a bill to protect PLHIV, however it has not translated it into effective implementation.^36^ For the other 3 provinces, legislation does not directly protect PLHIV from discrimination at work or in acquiring work. According to a recent report by United Nations Development Programme (UNDP), there might be some protection available to PLHIV under the Disabled Person Ordinance, 1981, as it reserves at least 1% of employment within a province for people with some disease or health condition. Yet, it is unclear if PLHIV benefit from the Disabled Person Ordinance.^37^ HIV treatment and prevention efforts are further complicated by Pakistan’s recent experience with devolution, in the presence of a weak health system.^38^ In 2011, the Ministry of Health was dissolved and health policy formation and planning was devolved to the provinces (Punjab, Sindh, Balochistan, Khyber Pukhtoonkhwa). With devolution in 2011, HIV program administrators have had to perform expanded functions, such as developing their own context-specific strategies and action plans.^39^ Other challenges faced by the health system include, a weak coordinating authority at the federal level, capacity and implementation constraints, and inadequate interprovincial information sharing.^40^ Pakistan also suffers from low overall health system capacity. Health in general remains a low priority for the government- the government of Pakistan spends approximately 0.5% of its gross domestic product (GDP) on health.^41^ In 2013-2014, total health expenditure was Rs. 757 billion ($6.2 billion), out of which Rs. 243 billion ($1.99 billion) was spent by the public sector and Rs. 508 billion ($4.17) by the private sector.^42^

In terms of global pressure, HIV is a sector with heavy foreign involvement. International donor organizations are the largest source of revenue for HIV prevention, treatment and care in the country. The involvement of major donor organizations has changed over time based on shifts in the donor landscape. From 2003-2009, HIV program activities were shaped by the World Bank, through a partnership between the World Bank and the national and provincial HIV programs (the Enhanced HIV/AIDS Control Program).^43^ With the withdrawal of the World Bank in 2010, the Global Fund^ [ 1]^ became the main financer of HIV related efforts. In 2015 it committed approximately $27.7 million for HIV efforts in Pakistan for 3 years. The highest spending is on prevention activities (IDUs are the largest focus of HIV prevention activities),^33^ followed by care and treatment. Services to HIV high-risk populations are provided through collaboration between the implementing partners of the Global Fund,^44^ which for Pakistan are the national AIDS Control program, the provincial AIDS Control programs, and NGOs.^45^ NGOs are hired on contracts by the provincial AIDS Control programs through a competitive bidding process.^46^ These NGOs provide needles to IDUs through needle exchange programs and in doing so are at the forefront of harm reduction interventions in the country.

Consequently, local forces (the politically contentious nature of HIV, the country’s recent experience with devolution, and high HIV disease burden among IDUs) and international forces (Pakistan’s place among the high impact HIV countries, donor funding for HIV warranting results, and increased global pressure to meet new targets) have created an environment that can exert an opposing pull on HIV service provision. The interplay between local and international forces makes it interesting to study the types of challenges HIV programs in Pakistan face in achieving the new 90-90-90 targets.

## Methods

### Data Collection

#### Participants

Twenty-nine individuals were interviewed (23 semi-structured, key informant interviews and 2 group interviews) between January 2015 to February 2015. Respondents were health and HIV experts in international organizations, and government employees from the national AIDS Control Program and provincial AIDS Control Programs in 3 provinces: Punjab, Sindh, and Khyber Pukhtoonkhwa. These respondents were selected as they were involved either in HIV policy formation, program management or implementation. Due to security concerns of traveling in Balochistan to conduct in-person interviews, 2 interviews with HIV program staff were conducted by phone, and more interviews were not arranged. Interviews could not be arranged with the Ministry of Narcotics Control, an organization whose mandate focuses on controlling the use and circulation of illegal drugs in the country.^47^ Parliamentarians were not interviewed as they were not involved in HIV policy formation or implementation.

Respondents from HIV programs were recruited by contacting senior management in the health department, who subsequently referred us to a key informant within the HIV program. Further recruitment of respondents within HIV programs took place through snowball sampling. The number of key informants (that is, individuals who focused on policy formation or implementation) within each HIV program informed the sample size for each HIV program. While all respondents in HIV programs had a general understanding of the challenges faced by the programs, however, based on their position in the program, some were able to elaborate more on the day-to-day challenges faced by the programs, while others on the broader policy aspects. Taken together, their responses helped in building a richer and more comprehensive picture of the challenges faced by the programs. Respondents for international organizations were recruited by contacting senior management in the organizations, and subsequently being connected to the health or HIV expert within the organization. The number of key international organizations in the HIV landscape determined the selection of international organizations (See Table).

**Table:** Breakdown of Interviews

**Respondent Type**	**Mode of Inquiry**
Federal government	
National AIDS Control Program	2 in-person semi-structured interviews
Provincial governments	
Punjab AIDS Control Program	5 in-person semi-structured interviews
Sindh AIDS Control Program	5 in-person semi-structured interviews; 1 in-person semi structured group interview (4 participants)
Khyber Pukhtoonkhwa AIDS Control Program	1 in-person interview; 1 email interview; 1 in-person semi structured group interview (6 participants)
Balochistan AIDS Control Program	2 phone interviews
International organizations	5 in-person semi-structured interviews; 2 phone interviews with respondents from 6 international organizations

Source: Authors own tabulations.

#### Interview Procedures

One researcher (the first author) conducted all interviews. The interviews lasted between 45 to 90 minutes. They were audio taped and transcribed verbatim where the interviewee consented, and in other instances field notes were taken. Seven interviewees, one from an international organization and 6 from HIV programs, did not consent to being recorded, and so field notes were taken. We had 2 interview guides, one for international organizations and another one for the HIV program employees. The interview guides were developed to generate conversation^48^ and focused on the strengths and challenges faced by the HIV programs. The interview guide for international organizations focused on understanding the scope of the organization’s involvement with HIV, political commitment for HIV across the provinces, and factors contributing to the concentrated epidemic in country. The interview guide for HIV program employees focused on understanding policy evolution, policies towards risk groups, needle exchange programs, experience working with NGOs, political support, financing arrangements, and factors contributing to the concentrated epidemic in country. As this paper is part of a larger research study, while the interview guides touched on a variety of policy and program areas, some of them are not included in this paper (See interview guides in Supplementary file 1)

#### Human Subjects Protection

Participation in interviews was voluntary and identities were anonymized for confidentiality. Prior to the interviews, the objective of the research was explained to all participants, and consent for tape recording was obtained. The University at Albany’s Internal Review Board reviewed the interview guide and deemed it exempt as respondents were official representatives of HIV programs who were answering questions in their official capacity.

### Data Analysis

We used an inductive interpretivist approach to coding the transcripts and field notes, whereby codes emerged from the data and were not determined in advance.^49,50^ First, all transcripts were open coded by reading each transcript line by line and then assigning codes to sentences and paragraphs.^51^ These codes reflected a theme or an idea that was being expressed in the text. The coding process was iterative and repeated for each interview. After open coding all transcripts, the codes from each interview were reviewed and consolidated into broader, more meaningful categories to form sub-themes, and from them common themes were formed.^52,53^ The first author coded the data, and the second author reviewed the codes. The first author discussed the codes, themes and sub-themes with the second author for data validation.^54^ Findings from the interviews are triangulated in the discussion section of the paper using information from newspapers and existing academic literature.

## Results

Key informants described challenges to meeting global HIV targets, which we categorized into 2 overarching themes of political challenges and governance challenges (see Box 1). Political challenges encompassed factors related to the degree of “political commitment” of country leadership and the policy and legal environment in which those charged with implementing programs operated. We understood “political commitment” to constitute 3 interrelated components, expressed, institutional and budgetary commitment. Expressed commitment refers to declarations of public support for HIV by prominent personnel in a country. Budgetary commitment is related to allocations for HIV programming, while institutional commitment focuses on the establishment of AIDS bureaucracies, adoption of policies, legislation, and HIV surveillance and monitoring systems.^55^ Governance challenges encompassed factors related to the service delivery environment including health system management, resource shortages and state-NGO relations. We understood “governance” as factors influencing decision-making procedures, rules and norms, in a given policy domain, around which managers converge that affect the implementation of policies.^56^

**Box 1. Factors Influencing Achieving the 90-90-90 Target for IDUs and Their Interconnections**

**Overall policy environment**

- Low health budget
- Pakistan is a drug route for Afghanistan and so a large number of drugs and drug users
- Injecting drug use and sex work are illegal in the country. Maternal and child health, and other diseases receiver greater attention from the government
- Needle programs are currently operational in the country, but fall in the ambit of prohibited practices
- Opioid substitution therapy is banned in the country

**
Governance challenges
**

- Limited healthcare infrastructure, poor regulation and management across the health sector
- Resource mismanagement and shortage
- NGO-state relationship management complicates service provision

**
Political challenges
**

- Limited & heterogeneous political commitment for HIV
- The conservative legal environment adds complexity to treating IDUs due to the ban on opioid substitution therapy

Abbreviations: IDUs, injecting drug users; NGO, non-governmental organization.

### Political Challenges

#### Low Political Commitment Hinders Budget Allocation and Support for Needle Exchange Programs

According to most respondents, political commitment was an important factor in ensuring the success of health programs as it determined which program was financed, programs with higher political commitment were more likely to get funded. The views of most respondents can best be summarized as:

*“If the political leadership is committed then you can get financing for the program from the Chief Minister and the Finance Department. If you do not have a strong liaison with Finance Department, and the Planning and Development department, then you cannot get resources”* (Government employee).

Almost all respondents reported low political commitment for HIV and gave examples of expressed and budgetary commitment to express low political commitment. To quote one respondent on low budgetary commitment:

“*The government has no commitment as such. A government is committed when it creates fiscal space for something. I would say it is not even committed to health, so HIV is really down in that list” (International organization).*

Almost all respondents in international organizations and across government departments discussed several reasons for low HIV support, which hampered their ability to effectively respond to the disease in ways that would be likely to further international targets. These included, a shift in global focus away from HIV, the lack of a champion for HIV, and stigma and discrimination, which hampered political will to prioritize this issue. Respondents also discussed low HIV disease burden in the general population and HIV being in competition with other diseases and health conditions as factors that impeded attention to the disease. They explained that the government was especially focusing on improving maternal and child health and polio indicators, as Pakistan is now one of the very few countries that has been unsuccessful in eradicating polio or providing a nurturing environment for infants and mothers. Drawing comparisons between HIV and other health conditions, one respondent remarked:

*“There is no political commitment, the focus is on polio and nutrition. [..] We have done a lot of advocacy in the Ministry, but the Planning Commission is saying that we don’t consider it important”* (Government employee).

Given the recent decentralization efforts, political commitment was also variable across the provinces. For instance, respondents also discussed how differences in political commitment across the provinces affected the use of needle exchange programs. While needle exchange programs were being implemented in Punjab, Sindh, and Balochistan at the time of the interviews, Khyber Pukhtoonkhwa did not have a needle exchange program. A few respondents in the HIV program of Khyber Pukhtoonkhwa highlighted that this was due to the conservative nature of the Pathans^ [2]^ that the program could not be implemented. In terms of differences in political commitment across the provinces for HIV programs, all HIV experts in international organizations ranked Punjab as having the highest commitment to HIV, followed by Sindh, Khyber Pukhtoonkhwa, and Balochistan. According to one respondent:

*“Punjab would be first, because as much as Sindh does realize the issue, it is not a priority because they have other priorities. Balochistan, well, someone from a donor organization* ^[3]^ *wrote to them and said that we will give you the money and you use it on your health. They said, we don’t need the money, we need to build roads. So Punjab, number 1, Sindh, number 2, KPK, number 3, and Balochistan, number 4”* (International organization).

To triangulate the ranking of political commitment for HIV programs across the provinces by international organizations, government HIV program respondents were asked to report their own perception of the political commitment for HIV in their respective provinces. To give an example of political commitment, all HIV program respondents in government departments (across Punjab, Sindh, and Khyber Pukhtoonkhwa) referred to the approval of the financial document, the Planning Commission Form 1 (PC-1) – a work plan document with cost estimates for a project – to signal government support for the program. For Punjab and Sindh, this document had been approved while for Khyber Pukhtoonkhwa it was still pending. Additionally, a key respondent in the HIV program in Khyber Pukhtoonkhwa highlighted that HIV was a very low priority for the government as it was focusing on a reform of the health sector, and it was plausible that the funding for HIV would be diverted towards these reform efforts. Information on how successful the approval of the PC-1 in Balochistan had been could not be obtained.

#### The Conservative Legal Environment Adds Complexity to Meeting the 90-90-90 Targets

The low political commitment of the government to HIV was also visible in the policy environment in which program implementers worked where the “institutional commitment” of the government was weak. Needle exchange programs are tolerated, although not supported by legislation. However, according to several respondents, opioid use is criminalized in Pakistan and faces harsh penalties as like sex work it falls under the category of addictive un-Islamic behavior. To quote one respondent:

“* Injecting drug use is illegal, because it is a form of addiction and sex work is also illegal here in the Islamic Republic of Pakistan” (Government employee).*

A few respondents discussed that it was important for HIV programs to take the religious environment of the country into consideration while devising policy and program activities. Giving examples of the importance of religious groups in influencing program decisions, one respondent highlighted that while needle programs and condoms prevent HIV transmission, religious groups were not in favor of such programs as they assumed that they could lead to an increase in HIV transmission. Another respondent remarked that efforts to change the legal environment surrounding sex work in Pakistan, or bringing too much attention to current service interventions for sex workers, could have negative consequences for HIV programs and should be avoided. According to the respondent:

“* There are certain things that should be left the way they are as they allow you room to work. So far, no one has not allowed us to work with FSWs [female sex workers], to provide condoms or tell them, ‘this is how you should go about it.’ But, just saying that they are FSW, half the maulvis^ [4]^ will be out on the street telling you not to do this. [..] So far the work is being done, the government knows but society doesn’t know, and let it happen. But, once they find out that this is happening then they won’t even let this happen. So, it is more dangerous to address and start flagging those issues” (International organization).*

As with sex work and needle programs, legislation does not support the use of opiate substitution therapy in Pakistan. The 90-90-90 targets stipulate that 90% of all PLHIV receive ART, and achieve viral suppression by 2020, but in order for viral suppression to be reached, adherence to ART is key. Yet, respondents described that physicians were reluctant to treat active drug users over a concern that irregular medicine use could lead to the development of a resistant strain of the virus. Most of the government respondents highlighted that both the increase in ART coverage and physician concerns of adherence to ART could be simultaneously achieved if the use of opiate substitution therapy (a replacement drug which is administered in clinical settings to counter opiate dependency among drug users) is introduced in Pakistan. They added that while, world over, the use of opiate substitution therapy has been shown to improve adherence to ART, the Ministry of Narcotics Control has banned the use of opiate substitution therapy, despite advocacy by HIV program employees, due to concerns of drug pilferage and for introducing a new drug in the market. Drawing parallels with Iran, a country which like Pakistan has a huge IDU population, one respondent remarked:

*“They are saying that this drug will slip, and there will be leakage in the market. We tried to convince them that 600 000 people in Iran are on methadone treatment […] but our Ministry doesn’t agree with it”* (Government employee).

Consequently, HIV programs focus on providing treatment to IDUs living with their families, as they are easier to track and exclude active drug users, who are street-based, from treatment. According to one respondent:

“*You have all IDUs, and so I can’t even put 40% of my patients on treatment. UNAIDS/WHO guidelines for 2016 are, treatment for all. In Pakistan as we have a high proportion of IDUs we can’t really implement this” (Government employee).*

Therefore, according to the respondents a large portion of IDUs are excluded from treatment and Pakistan cannot comply with the ‘prevention for all’ treatment guidelines.

### Governance Challenges

#### Weak Health System and Financial Management

Almost all government employees provided examples of poor regulation and management across the health sector, and discussed the harmful consequences associated with it. Most respondents highlighted that ineffective blood screening and poor control of hospital infections posed a challenge for the health system, including HIV. Highlighting the importance of taking a horizontal versus a vertical approach to addressing HIV, one respondent remarked:

*“HIV is not an island and so the work I do is not done in isolation [….] When your entire health system functions well, then every communicable disease will be controlled, and HIV will be controlled and will be part of that”* (Government employee).

A few respondents highlighted that low health system outreach and capacity had led to an expansion of unregulated private sector providers, called ‘quacks.’ These unregulated providers increased the overall level of risk in the system as they often did not take the required safety precautions. Respondents explained that while there have been several positive developments such as efforts to control quackery, and a disposable syringe act in Sindh, to control the re-use of syringes, they had not been enforced due to low regulatory capacity. Referring to one respondent:

*“In Jalalpur Jutta, a quack was using unsterilized syringes and this led to a massive outbreak of HIV/AIDS”* (Government employee).

A few respondents in government departments expressed concerns regarding the spread of HIV from the IDUs to their spouses and one respondent remarked that it was the outcome of poor linkage to care. In terms of resources, a few respondents described that HIV resources were only sufficient to provide services to a very small portion of the IDUs, which was preventing the expansion of program activities. Quoting one respondent:

*“Theory says that if you want to control your epidemic then you have to give prevention services to 80% of the high risk group, now through Global Fund Support we just reach 17% of IDUs”* (Government employee).

While a few respondents discussed insufficiency of funds, almost all respondents in government departments and several respondents in international organizations discussed that a more substantial obstacle to meeting targets, as compared to the availability of resources, was resource mismanagement. Examples of mismanagement included misplaced resource allocation priorities and a lack of spending capacity, a problem experiences by government departments more generally, due to which funds get lapsed. To quote one respondent:

*“Resources are not an issue. I work here, I know resources are there. Utilization of resources is poor. We will use Rs. 400 000 on a meeting and will not spend on a center. So there is a need to fix direction”* (Government employee).

As the quotation above implies the amount of resources available for HIV were not in short supply. However, the processes by which decisions were made on how to allocate funds were not made in a rational or transparent manner.

Following a discussion of the weaknesses in the current health infrastructure, most government employees explained that post devolution, there was a need to strengthen the health infrastructure through better regulation and management and by developing capacity for planning and implementing policy. A few respondents highlighted that frequent transfers of government employees, hampered effective monitoring of operations, as by the time a government employee understood the intricacies of the program, he/she was transferred to a different location. They also pointed to a need for program evaluation, arguing that all the emphasis was on preparing program reports with little attention being given to evaluating the effectiveness of programs.

#### Challenges in Governing State–Non-governmental Organization Relations

Harm reduction services for both IDUs and other risk groups are delivered through NGOs hired on contracts. All government respondents regarded NGOs as essential for HIV programs for 2 main reasons. First, almost all respondents highlighted that NGOs have extensive outreach in the HIV high-risk communities, as many NGO workers are also ex-drug users and sex workers. Service delivery through NGO workers is preferred as NGO workers know the hot-spot areas and are also perceived as being from within the communities. Consequently drug users and sex workers do not hide from them, enabling service delivery. In contrast, the government is considered an outsider who is responsible for criminalizing their behavior and so they hide from the government. Second, a few respondents highlighted that as drug users and sex workers are punishable by law and so the government cannot openly provide them with HIV program services. AIDS programs expect to face resistance from politicians and religious communities if the government is seen providing services to these populations. According to one respondent:

*“One of the main issues we faced was that can we give services to populations that are considered illegal? As a government, can we do that? No, we cannot, and so we chose to go through the NGOs. The second thing is that these are hidden populations and they do not want to come in front of the government. [..] The other thing is that these NGOs are deep-rooted, and are very close to the communities. They are ex-sex workers, ex-drug users and have very close relations with these populations”* (Government employee).

While the preceding discussion highlights that NGOs are essential for the government for effective service delivery, almost all respondents who described the importance of NGOs for the government also expressed concern regarding ineffective use of government funds by NGOs, given the nature of the partnership. A few respondents expressed concerns that as the same NGOs were repeatedly hired for delivering services, they had learnt how to game the system. They argued that the same work could be done more cost effectively by other newer actors, if given a chance. The views of respondents can best be summarized as:

“*HIV is not a pleasant sector. There are a lot of conflicting interests. This is not a selfless type thing that a nun comes and controls leprosy. This is a public-private partnership and so there are a lot of interests. I am sitting here as a government employee, so I will want a good outcome but the other person may not want the same. When you have an NGO and I have to implement my work through them, the well being of the patient is inversely proportional to the well being of the organization, unfortunately. This is not how it should be, but it is. As much importance you give to the organization, that much the patient will be neglected. Needle exchange programs are good, but when I see the budgets, sometimes, I feel like laughing that how can an outreach worker distribute 100 syringes in one day? So if he says to me I went to x-y-z places, some places are just not possible” (Government employee).*

While interviews with government employees highlighted the importance of the NGOs for the government and the relationship challenges, a few respondents from international organizations described that the relationship between NGOs and the government was strained, and often created a hostile environment for both sides. According to one respondent:

“*This is a problem at both ends and so you cannot blame only one side. [….] Majority of NGOs that work in province x*^ [5]^ *[…], they join hands together against the government […] The government sometimes with a stroke of the pen says that we will finish your funding […].”* (International organization).

In the quotation above the respondent is ascribing responsibility for the tense relationship between NGOs and the government to both actors.

## Discussion

Using Pakistan as a case, we explored the challenges to the implementation of HIV programs that are likely to hinder the country’s ability to meet ambitious goals such as the 90-90-90 target. We found both political and governance challenges to achieving the target, particularly in the context of the global HIV scale-down. Political challenges included, low support for HIV and a conservative legal environment that contributed towards the ban on opiate substitution therapy creating low treatment coverage. Governance challenges included poor regulation and management of the weak health infrastructure, resource mismanagement, and strained state-NGO relations.

Recent literature on health system strengthening emphasizes the importance of governance,^57^ develops a number of frameworks for assessing governance,^58-60^ and highlights a positive relationship between governance and health outcomes.^60,61^ Studies have shown that effective governance of health sector organizations can lead to better implementation and uptake of health technologies.^62^ Higher regulatory quality and strong public sector financial management, core components of governance,^63^ are associated with lower infant mortality rates,^64^ reduced corruption , and increased public health spending.^65^ However, despite the widely established benefits of governance, LMICs suffer from weak institutions, poor public infrastructure, low legal capacity,^66^ and weak accountability mechanisms.^67^ The need for domestic governance is further exacerbated by inadequate governance of the international response to HIV.^68^ Governance of intellectual property rights influences access to medicines in LMICs,^68^ yet recent evidence suggests that PLHIV in LMICs do not have access to high quality drugs.^15^ Similarly, the mismatch between donor and local priorities^11^ can partially be attributed to the inability of LMICs to participate in decision-making at the global institutional level.^68^ Finally, a number of stakeholders working on a variety of externally driven health initiatives,^69^ including HIV, has burdened governments in LMICs who do not have the capacity to manage multiple programs, suggesting a need for better harmonization of policy responses.^70^ Our findings support existing literature on the need for better governance across LMICs, as we identify weak governance of the health system, poor resource management,^38^ and a need to regulate informal health providers in Pakistan.^71,72^ Our results also highlight the importance of developing global targets that take local contexts and political factors into consideration, as the ban on opiate substitution therapy prevents implementation of the ‘global treatment for all’ policy target. Our findings add to the literature on health governance by providing evidence of a need to improve governance of contracts. While research on governance in health emphasizes strengthening health systems^73^ and leadership,^74^ health system governance frameworks devote little attention to exploring and emphasizing the role of non-governmental actors in governance.^75^ Yet, changing government structures^76^ call for greater governance of state-NGO relations, as unequal power sharing and competing ideologies can lead to hostility and mistrust between these actors.^77,78^ Consistent with another study on Pakistan, which identified a tense state-NGO relationship,^46^ we find a lack of trust between NGOs, hired for delivering services to HIV risk groups which can prove to be detrimental for service provision.^79^

We also find support for the importance of political commitment in influencing program outcomes. Political commitment is important for mounting a successful response to HIV.^55^ Higher political commitment is linked with budgetary authorizations,^80-82^ laws that support distribution and use of needles,^83,84^ and adoption of service delivery programs.^83-85^ Our respondents discuss political commitment as a determinant of funding allocations, for bringing an item on to the policy agenda, and also for getting drug laws approved. Our findings highlight that while Pakistan did adopt a relatively effective needle exchange program across Punjab and Sindh, despite the absence of legislation supporting such a program, it has been unsuccessful in implementing ‘treatment for all’ due to the ban on opiate substitution therapy.^35^ More generally, overall low political commitment for HIV, a heavy reliance on donor funding, delays in budget approval, and budget preferences for other diseases highlight that politics plays a major role in the effective functioning of the health system in Pakistan.^86^ Our findings also highlight that in addition to political support from the government, program managers also have to be wary of different interest groups in the country while devising policy and program activities. Interest groups, especially religious groups, have been shown to influence public policy across countries.^87^ Our findings support this existing literature as our respondents were sensitive towards the sentiment of religious actors in the country. More specifically, HIV programs distance themselves from service provision by choosing to deliver services through NGOs, and avoid drawing unnecessary attention to HIV program activities to avoid backlash from religious groups and the other civil society actors.

There are several implications of our findings. Our findings point to the importance of governance and political commitment for achieving the 90-90-90 target. They highlight that local contexts make it difficult, if not impossible, to achieve global goals. Several suggestions from our respondents such as effectively engaging the government, and creating champions^88^ to garner support for HIV can help in increasing political commitment towards HIV. Other strategies to build political commitment can include collaboration and coalition building across different government sectors and with actors outside the government^60^ through repetition and reinforcement of AIDS prevention and treatment messages. More recently, big data and biomedical approaches have been promoted to garner support. However, it is important to be aware of the benefits and limitations of such approaches. For example, variation in data quality and coverage, reaching hidden populations, procedures governing data sharing and transparency, all point towards a need to be critical towards an overreliance on big data. Similarly, excessive bio medicalization of HIV, has pushed important challenges of dealing with HIV, such as HIV stigma into the background.^24^ Our findings also imply a need for improved governance, especially in context of the current global scale down of HIV funding. Given the strained state-civil society relationship, they highlight the importance of tailoring the style of governance to match changing service provision structures. Our findings suggest that as governments move towards delivering services through contracts, they will need to re-think which combination of the 3 modes of governance – authoritarian, transactional or persuasion^ [6] ^– is most suited to achieving effective service provision.^89^

Our study has several limitations. We were only able to interview 2 respondents from the Balochistan HIV program due to security concerns, and were unable to arrange interviews with NGO outreach workers. There was only one interviewer and coder for the study and this could lead to researcher bias, however, an effort was made to overcome this through a thorough review and discussion of codes and sub-themes by the second author.

## Conclusion

This study aimed to explore how different policy actors were navigating the challenges posed by the evolving global HIV response in a country with a conservative climate towards HIV, a large IDU population, and competing health sector priorities. We identified political and governance challenges that are likely to hinder target achievement. Our findings suggest a need invest in improving governance, and to focus attention on collaboration and coalition building across different government sectors and with actors outside the government. Future work can explore the relationship between different styles of governance and health outcomes.

## Acknowledgements

The authors are grateful to Dr. Patricia Strach and Dr. Erika Martin for comments on an earlier draft of the paper.

## Ethical issues

The University at Albany’s Internal Review Board reviewed the interview guide and deemed it exempt as respondents were official representative of HIV programs who were answering questions in their official capacity.

## Competing interests

Authors declare that they have no competing interests.

## Authors’ contributions

Conception and design: HK and AMF. Data collection: HK. Analysis and interpretation of data: HK and AMF. Drafting of the manuscript: HK. Critical comments and editorial review: AMF. Supervision: AMF.

## Endnotes


^[1]^ A donor agency with the mandate to attract, manage and disburse resources through a public private partnership for HIV/AIDS, tuberculosis and malaria in developing countries.



^[2]^Ethnic community who primarily resides in the province.



^[3]^We have removed the name of the organization to maintain confidentiality.



^[4]^Maulvi’s is the local term for religious leaders in Pakistan.



^[5]^We have removed the name of the province to maintain confidentiality.



^[6]^The authoritarian mode of governance is focused on command and control, the center exercises power and expects compliance. In the transactional mode of governance, more common with contractual arrangements, performance frameworks and targets are set. In governance by persuasion, a shared vision is created, there is horizontal accountability and shared goals and standards.^89^


## Authors’ affiliations

^1^Department of Economics, School of Humanities and Social Sciences, Information Technology University, Lahore, Pakistan. ^2^Rockefeller College of Public Affairs and Policy, University at Albany, Albany, NY, USA.

## Supplementary files


Supplementary file 1. Interview Guides.

## Key messages

**Implications for policy makers**

- Epidemic control will be difficult to achieve as long as there is poor governance of the health system and low political support for HIV.
- For successful service delivery through contracts, the government and civil society will need to work towards improving the governance of contracts.
- Taking a more horizontal approach to service delivery, versus focusing on vertical programs, can help in improving health outcomes with reference to HIV and general health.

**Implications for public**

With waning global attention to HIV, vulnerable populations face increased obstacles to receiving care and treatment as governments fail to prioritize HIV and the behaviors that fuel it. Injecting drug use, the main mode of HIV transmission in the country is treated as a criminal act rather than a medical condition. Reduced HIV stigma and discrimination, through greater awareness and advocacy, can help people living with HIV (PLHIV) lead lives as equal citizens of society.
