# Supplementary material for: Political and Governance Challenges to Achieving Global HIV Goals with Injecting Drug Users: The Case of Pakistan
Source: Int J Health Policy Manag. 2019 Jan 22;8(5):261–71. doi: 10.15171/ijhpm.2018.131 (PMC6571491; doi:10.15171/ijhpm.2018.131)
Supplement: Supplementary file 1 — Interview Guides. [file ijhpm-8-261-s001.pdf]

Supplementary file 1. Interview Guides

Interview guide for international organizations

1) Explanation of the purpose of the interview; discuss informed consent and confidentiality; get verbal permission to tape record.
2) Grand tour question
   - What is your position title and can you briefly describe your main duties?
   - For how long have you been at the organization?
3) Understanding HIV/AIDS from the perspective of the international organization

   Understanding the policy over all

   [From a birds eye view perspective, lets first talk a bit about the over all HIV/AIDS policy]
   - What is your understanding of the main features of the HIV/AIDS policy of the government in the provinces you work in?
   - Can you tell me how this policy evolved?
   - There were a number of stakeholder involved in forming the policy, so was there a lot of disagreement?
   - Where is this policy going? Are there some changes on the horizon?
     - Probe: Do you think the policy is oriented towards treatment verses control? One risk group verses another?
   - Do you think there should be a focus on something and it is not?

   Understanding the scope of the organizations involvement
   - Which provinces do you work in?
   - Would you consider yourself to be a main actor in the HIV/AIDS field
   - What are your interventions and who are your interventions targeted towards?
     - Probe: What types of projects are you more focused on? [advocacy , treatment, care, capacity building]
     - Probe: Are you involved in the harm reduction activities with NGOs or the government in any way? Different risk groups – SEP programs, IDUs, sex workers? [Probe: Ask about tamer project for DFID - this was a capacity building project for NGOs.]
   - How was it chosen to target the policy towards (group they mention)?
   - Do you have input in the overall HIV policy formulation? Implementation? Program expansion? Of provinces?
   - What are some of the challenges you face in terms of implementing the policies/programs? How are they similar / different across the provinces?

   Devolution
   - How did the scope of your activities change following devolution?
   - Who do you mostly coordinate with now?

   Financing
- Can you walk me through how the funding process works?
  - Do you provide financing for HIV/AIDS?

- What do you provide the largest share of funding for? [type of activity; risk groups]
- What do you provide the smallest share of funding for? [type of activity; risk groups]
- Who decides how much funding will go where?
- Does disbursement of funding involve discussion with the receiving agency or do you have set priorities and goals that you have to meet?

To get at budgetary commitment

- Compared to other programs you fund, how would you compare HIV/AIDS funding?
- Who do you provide the highest share of funding for, and in what province mostly?
- Please can you share these estimates with me?
- What are some of the factors that made you allocate money in this way?

To get at expressed commitment

- Have political leaders in the provinces shown support for HIV/AIDS program – your efforts?
- How has this changed over time?
- Have they done something to support one risk group more or less than another?
- Which disease do you think they give most attention to?
  - Which of the following do you think they consider bigger problem. Rank the following in order of importance (1 most important; 5 least important): HIV/AIDS; viral hepatitis; tuberculosis; maternal and child welfare; Condition overall healthcare system
- Would you also rank them did you order them in this way?

To get at institutional commitment

- Do you think there is institutional commitment across the provinces for addressing HIV/AIDS?

Causes of HIV/AIDS increasing

- What would you list as being some of the main reasons behind why HIV/AIDS is increasing in the country? [Probe: media attention, system failure; stigma, nature of the epidemic – it is concentrated among IDUs, HIV/AIDS is sensitive]
- Do you think one risk group gets more preferential treatment as compared to the other?
- What do you think about the media campaign regarding HIV/AIDS? Is there even a media campaign?
  - Do you think that the media has done it’s due part in creating awareness about HIV/AIDS? Have there been any media campaigns in the recent past?

What do people think in general

- Working in the field, or through your general knowledge do you think the general population perceive someone living with HIV/AIDS in a negative or positive light? Do they perceive risk groups in the same way? Different way?

- Is there something in the HIV/AIDS policy that really bothers you? You would like to change?
- What do you like about the way HIV/AIDS policy works?
- So we keep hearing that aid has not been effective, why do you think this is the case?

Policy advocates and champions

Is there a high level advocate or champion who promotes HIV/AIDS issues?
Are there any civil society groups that promote HIV/AIDS issues?

4) **Conclusion**
    - Before we finish our discussion, do you have any other comments about the HIV/AIDS program that we have not yet discussed?
    - Are there any individuals who you think we should talk to about this?
    - I hope that we can get back to you with additional questions if they arise. Is that okay with you?

Interview guide for government employees

Title: Understanding the policy response to HIV/AIDS in Pakistan
PI: Hina Khalid

1) Explanation of the purpose of the interview; discuss informed consent and confidentiality; get verbal permission to tape record.
2) Grand tour question

- What is your position title and can you briefly describe your main duties?
- For how long have you been at the department

Understanding policy evolution

1. What are the main features of the HIV/AIDS strategy of the province after 2011 (devolution)?
2. Can you tell me how this strategy evolved (compared to the strategy before devolution)?
3. Do you think your department is equipped to handle changes post devolution?
4. What are some of the issues you confront on a daily basis in policy implementation?
5. Is there some type of policy coordination between the national and the provincial government? Do both take some types of specific roles? Do you coordinate with other provinces?
6. Are there areas where you would like more input from the national government?
7. What role do donor organizations play?
    - Formulation? Implementation?
8. A number of stakeholders have been listed in formulating the HIV/AIDS strategy: NGOs, government officials, special working group, PLHIV
    - Was there a lot of disagreement in formulating the policy?
    - In your estimation, how cohesive would you say are the proponents of these policy solutions? [By cohesive I mean the degree to which the community agrees on the definition, causes and solutions to the problems]
9. Where is this policy going? Are there some changes on the horizon?

Broader umbrella

1. How does the current HIV/AIDS policy fit into the broader umbrella of HIV related disease care?
2. Is there more emphasis on HIV treatment verses prevention?

Target group

The AIDS strategy is targeted towards IDUs, sex workers, Maternal and children

1. Within these risk groups are there any that are perceived in a better way as compared to the other?
2. Is it more easier/harder to target policies and treatment towards one group as compared to another?

Syringe exchange programs

1. So, the policy with regards to injection drug users in the province involves Syringe Exchange Programs.
    a) Can I get some information on the number of syringe exchange programs that are currently in place and functional in the province?
    b) What is their size? Scope?

2. It appears that NGOs are the major stakeholders in delivering clean syringes to injection drug users.
    a) What are some of the challenges faced by syringe exchange programs?
    b) What are some of the successes of the syringe exchange programs?
    c) Can you please let me know who I can talk to better understand the functioning of SEP programs? Can you connect me to some NGOs?

<u>Other risk groups (sex workers)</u>

1. What is the policy with regards to sex workers? Please can you walk me through it's main features.
    - Is this also implemented through NGOS?
2. What are the main challenges faced in implementation/planning?
3. Do you think that it is easier or harder to target prevention policies towards these risk groups as compared to IDUs?
    a. What are some of the reasons for this?

<u>A. Budgetary commitment</u>

1. Does the government have a PC-1?
2. What is the overall resources/expenditure/budget available for HIV/AIDS interventions? Please can you share these estimates with me?
3. Can you please share them with me over time (going as far back as is possible)
4. Is it possible to rank order the risk groups from highest to lowest share of funding?
5. Can you walk me through how a decision is made to allocate money in this way?
6. Is it first by risk group and then by type of activity?

Funding

1. Who are the main donor organizations for HIV/AIDS funding?
2. Do you think international organizations are funding those aspects of HIV/AIDS that they should be financing?
3. Would you like them to consult you more or is this something they already do?

<u>B. Expressed commitment</u>

1. Have political leaders shown support for HIV/AIDS program?
2. If yes, why? If not why?
3. How has this changed over time?
4. Do all risk groups get equal treatment?
3. Is there some other disease that gets more attention than HIV/AIDS?
5. Please rank the following in order of importance (1 most important; 5 least important): HIV/AIDS; viral hepatitis; tuberculosis; maternal and child welfare; something else; Condition overall healthcare system)
6. Why did you order them in this way?
7. If the government had extra $ do you think they would spend on them in the order in which you have ranked them?

<u>C. Institutional commitment</u>

1. What are the number of programs that provide services to IDUs? To sex workers? Maternal and child health?

2. What were some of the factors that led to a difference in number of programs across risk groups?
3. Is the HIV/AIDS strategy part of the national health policy/plan?
4. Is the right to HIV/AIDS enlisted in the national legislation?
5. Is there some mechanism that coordinates HIV/AIDS programming?

Are/ How are policies coordinated between HIV/AIDS and Viral hepatitis, HIV/AIDS and Tuberculosis, HIV/AIDs and maternal and child health policies?

Focusing events and public attention

1. Have there been any major events in the last year that have drawn particular attention to HIV/AIDS problems in the country in the last few years?
2. How much attention do you think HIV/AIDS has received in the media?
3. Has the media played it's role in increasing awareness about HIV?
4. How would you compare this to Hep C? TB?
5. Are there any information campaigns for the general population? Who do they target?

Overall

8. What do you think despite all efforts HIV/AIDS is increasing in the country? What are some of the reasons why this might be the case?

Conclusion

1. Before we finish our discussion, do you have any other comments about the HIV/AIDS program that we have not yet discussed? Something you think I have missed out and should have focused on?
2. Are there any individuals who you think I should talk to about this?
3. I hope that we can get back to you with additional questions if they arise. Is that okay with you?
4. Please can you share (i) the AIDS strategy of the province, (ii) budgetary allocations for HIV/AIDS and (iii) any other written material that you think might be helpful?
